# A Proposed School of Hygiene

**Published:** 1887-03

**Authors:** 


A PROPOSED SCHOOL OF HYGIENE.—The University of Michigan is about to establish a school of hygiene to be placed under the direction of Prof. V. C. Vaughn of the State Board of Health. In this school besides the ordinary branches belonging to hygiene and sanitary science will be taught climatology, analysis of air, water, etc.

---

SEVERAL articles have recently been published in the journals maintaining the equine origin of tetanus. Dr. Dauvin, of the French navy, however, claims that he has seen the disease in Madagascar and the Antilles in places where there neither are nor ever have been horses.

---

THE death of Prof. Ludwig Bandl, formerly of Vienna, and recently Professor of Gynæcology in the University of Prague, has just been announced. Prof. Bandl attained a world-wide reputation by his study of the phenomena of uterine contraction.

---

THE homœopathists claim that sixty per cent. of Brunton's "hints" on therapeutics are derived from homœopathy. In Ringer's therapeutics only fifteen or twenty per cent. of his information was derived from the followers of Hahnemann.

---

UP to December 31, 1886, 1,726 patients had received preventive inoculation for rabies according to Pasteur's method, with fifteen deaths in France and thirty in the other countries.

---

MORE than eighty-one tons of quinine were used in the United States in 1885.

